# Mitochondrial Dynamics: Pathogenesis and Therapeutic Targets of Vascular Diseases

**DOI:** 10.3389/fcvm.2021.770574

**Published:** 2021-12-06

**Authors:** Yi Luan, Kai-Di Ren, Ying Luan, Xing Chen, Yang Yang

**Affiliations:** ^1^Department of Translational Medicine Center, The First Affiliated Hospital of Zhengzhou University, Zhengzhou, China; ^2^Department of Pharmacy, The First Affiliated Hospital of Zhengzhou University, Zhengzhou, China; ^3^Henan Key Laboratory of Precision Clinical Pharmacy, Zhengzhou University, Zhengzhou, China; ^4^Department of Physiology and Neurobiology, School of Basic Medical Sciences, Zhengzhou University, Zhengzhou, China

**Keywords:** cardiovascular disease (CVDs), vascular diseases, mitochondrial dynamics, fusion, fission

## Abstract

Vascular diseases, particularly atherosclerosis, are associated with high morbidity and mortality. Endothelial cell (EC) or vascular smooth muscle cell (VSMC) dysfunction leads to blood vessel abnormalities, which cause a series of vascular diseases. The mitochondria are the core sites of cell energy metabolism and function in blood vessel development and vascular disease pathogenesis. Mitochondrial dynamics, including fusion and fission, affect a variety of physiological or pathological processes. Multiple studies have confirmed the influence of mitochondrial dynamics on vascular diseases. This review discusses the regulatory mechanisms of mitochondrial dynamics, the key proteins that mediate mitochondrial fusion and fission, and their potential effects on ECs and VSMCs. We demonstrated the possibility of mitochondrial dynamics as a potential target for the treatment of vascular diseases.

## Introduction

Cardiovascular disease (CVD) is the leading cause of death worldwide (1). Vascular diseases, particularly atherosclerosis, are initiated at an early stage in life and remain asymptomatic for a long period until they reach advanced stages (2). Among vascular diseases, atherosclerosis is a pathologic process of lipid accumulation, scarring, and inflammation in the vascular wall, particularly the subendothelial (intimal) space of arteries, which leads to vascular wall thickening, luminal stenosis, and calcification (3). Endothelial cell (EC) activation or dysfunction is an early symptom of vascular diseases that occur at the lesion-prone sites of arterial blood vessels, where ECs display pro-inflammatory and prothrombotic phenotypes and reduced barrier function. Notably, ECs are extremely sensitive to oxidative stress and respond rapidly to altered environments, such as changes in oxygen levels, pathogen stimulation, and damaging endogenous stimuli (4). In addition, the direct contact between ECs and circulating immune cells triggers immune reactions (5). Another substance that plays an important role in blood vessel function is nitric oxide (NO). NO is a signaling molecule in the vascular system, in which blood vessels control blood flow by sending signals to the vessels to vasodilate. NO could also slow the deposition of atherosclerotic plaque on the blood vessel wall (6).

Other mechanisms and stimuli also affect the function of blood vessels. Blood flow promotes the production of adhesive molecules, which recruit inflammatory cells (7). Besides, the migration of vascular smooth muscle cells (VSMCs) also facilitates atherosclerosis progression (5). Several vascular diseases ultimately lead to myocardial infarction, stroke, and peripheral artery disease (8). The etiology of vascular diseases is complex; thus, several risk factors may contribute to their progression, including dyslipidemia, diabetes, smoking, hypertension, oxidative stressors, angiotensin II, systemic infection, and inflammation (9). Nonetheless, an effective cure for vascular diseases still lacks partially because of the complex etiology of the diseases in spite of recent advances (4).

**Figure 1:**
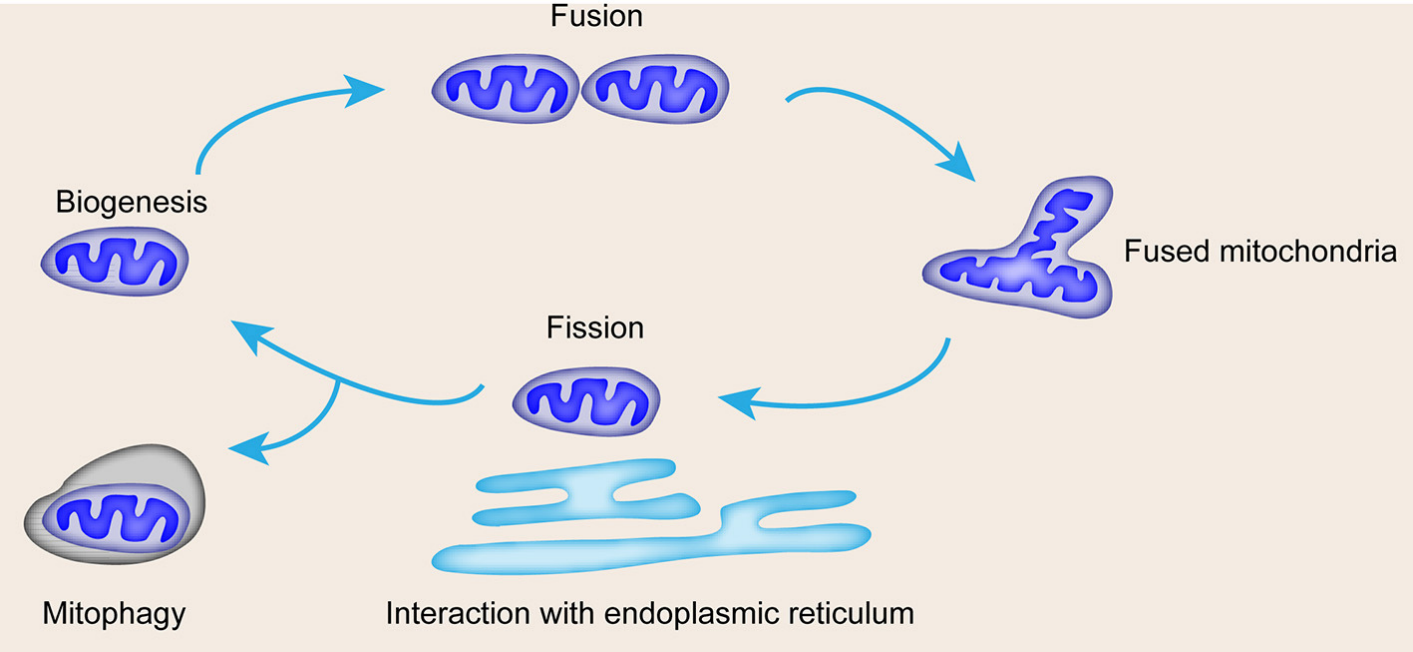
Mitochondrial life cycle and contribution of mitochondrial dynamics and mitophagy to quality control. Mitochondrial dynamics include biogenesis, fusion for mass increase, fission for number increase, interaction with ER, and mitophagy.

The occurrence of vascular diseases is related to the loss of energy metabolism; notably, the mitochondria are the core sites of cell energy metabolism (10). The mitochondria are important in endothelial and smooth muscle function (11, 12). Mitochondria are composed of a central mitochondrial matrix surrounded by two inner and outer mitochondrial membranes, and eukaryotic mitochondrial respiratory chain is composed of complex. Compounds I, II, III, IV and complex V (ATP synthase), ubiquinone, coenzyme Q and cytochrome C are located in the inner membrane of mitochondria. Mitochondrial respiratory chain oxidative phosphorylation is responsible for more than 90% of oxygen consumption and provides more than 95% of body energy. Supply, the mitochondrial matrix is the main site of the tricarboxylic acid cycle and fatty acid β oxidation. Apart from its capacity for ATP production, the mitochondria also modulate reactive oxygen species (ROS) generation, calcium regulation, cell death, and survival (13, 14). The function of the mitochondria is affected by mitochondrial dynamics, including fusion and fission, interaction with the endoplasmic reticulum (ER), and mitophagy (Figure 1). Mitochondrial dysfunction leads to cell senescence, inflammation, and apoptosis, which are characteristics of vascular diseases (15). In addition, mitochondrial dysfunction can be triggered by DNA damage, which is closely related to several risk factors of CVDs (16). In this review, we summarize the correlation between vascular diseases and mitochondrial dynamics with emphasis on the detailed function of mitochondrial dynamics in specific vascular disease forms and the potential therapeutic approach of mitochondrial dynamics in vascular diseases.

## Regulation of Mitochondrial Dynamics

More and more evidences indicate the role of mitochondrial dynamics in vascular function and the pathogenesis of vascular diseases. The mitochondria are highly dynamic organelles whose structure and distribution affect metabolism despite being recognized as isolated organelles (17). The nature of the dynamic network depends on the proper balance between mitochondrial fusion and fission (18). Its balance can be destroyed by environmental stimuli, developmental status, and cellular metabolic demands. The recently identified molecular mediators of mitochondrial fusion and fission, as well as post-translational modification (PTM) by an extensive set of kinases, phosphatases, and ubiquitination mediators, bring a new slight on the mechanism of mitochondrial dynamics (Table 1) (19).

**Table 1:** Mediators involved in the regulation of mitochondria fission and fusion.

**Mediator**	**Function in mitochondrial dynamics**	**Role of mediator in vascular diseases**
**Fusion mediators**		
Mitofusin-1	GTPase in outer mitochondrial membrane that tethers adjacent mitochondria	Atherosclerosis
Mitofusin-2	GTPase in outer mitochondrial membrane that tethers adjacent mitochondria	Pulmonary arterial hypertension, arterial restenosis, and atherosclerosis
Optic atrophy 1	GTPase in inner mitochondrial membrane that mediates fusion	
Fission mediator: DRP1	Cytosolic GTPase that translocates to the outer mitochondrial membrane when activated	Patent ductus arteriosus, pulmonary arterial hypertension, and atherosclerosis
**Fusogenic and fissogenic lipids**		
Phosphatidic acid	Generated by mitochondrial phospholipase D; promotes assembly of fusogenic mediators	
Diacylglycerol	Lipin-1, a protease that hydrolyzes phosphatidic acid, generates diacylglycerol, which promotes fission	
**Transcription factors**		
PGC-1α	Mediator of mitochondrial biogenesis and transcriptional coactivator of mitofusin-2	Pulmonary arterial hypertension
HIF1α	Hypoxic transcription factor that also promotes DRP1 activation and fission	Pulmonary arterial hypertension
**Post-translational regulators of DRP1**		
Cyclin B–cyclin-dependent kinase1	Serine–threonine kinase that initiates mitosis and also activates DRP1 by phosphorylation of DRP1 serine 616	Pulmonary arterial hypertension
Aurora A kinase	Serine-threonine kinase, regulating mitotic entry, chromosomal segregation, and DRP1 activation	
Calcium-calmodulin–dependent kinase	Activates DRP1	Patent ductus arteriosus
Calcineurin	Serine-threonine protein phosphatase that activates DRP1 by dephosphorylating DRP1 serine 637	
Protein kinase A	Causes cyclic AMP–dependent phosphorylation of DRP1 at serine 637, which inhibits fission	
SENP5	Moves to the mitochondria during mitosis and desumoylates DRP1, which leads to the activation of DRP1	

### Mitochondrial Fusion Proteins

**Figure 2:**
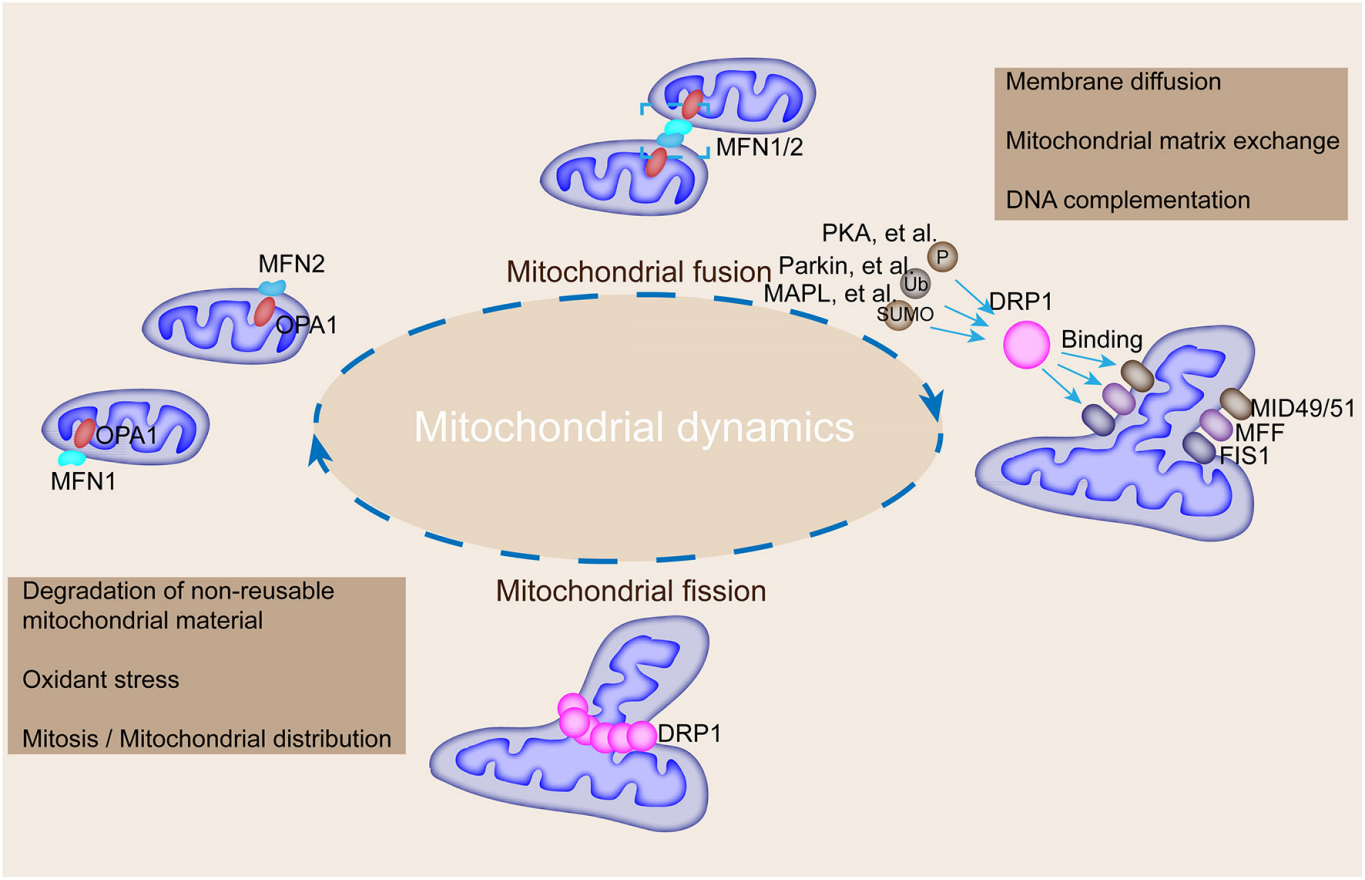
Mechanisms underlying the regulation of mitochondrial fusion and fission and their roles in modulating mitochondrial morphology. MFN1, MFN2, and OPA1 mediate mitochondrial fusion, whereas DRP1 interacts with FIS1, MFF, and MiD49/51 to participate in mitochondrial fission. DRP1 could be modified by phosphorylation, ubiquitination, and SUMOylation by corresponding enzymes to promote its binding with FIS1, MFF, and MiD49/51. Mitochondrial fusion is accompanied by membrane diffusion, matrix exchange, and DNA complementation, and fission is accompanied by the degradation of non-reusable mitochondrial material, oxidative stress, and mitosis.

Mechanically, mitochondrial fusion at the outer mitochondrial membrane is controlled by the transmembrane GTPases, MFN 1 and MFN2, and fusion at the inner membrane is controlled by optic atrophy protein 1 (OPA1) (20, 21). Besides, fission is regulated by DRP1 and fission-1 (FIS1) (22, 23). Mitochondrial fusion is regulated by the coordinated action of conserved GTPase proteins, including MFN1 and MFN2, and these transmembrane GTPases located in the outer membrane of the mitochondria are responsible for the regulation of the mitochondrial fusion by forming homodimeric or heterodimeric, antiparallel, coiled-coil linkages between adjacent mitochondria and C-terminal domains (24) (Figure 2). MFN1 and MFN2 deficiencies lead to a remarkable decrease in mitochondrial fusion (25). Additionally, MFN2 mediates cell apoptosis and mitochondrial autophagy (26). OPA1, a dynamin-related GTPase embedded in the inner membrane or intermembrane of the mitochondria, is involved in mitochondrial intima fusion and mitochondrial cristae remodeling (20). OPA1 harbors two forms (i.e., long and short OPA1 proteins) with distinguished functions (27). The long form of OPA1 located in the inner membrane, which is responsible for intimal fusion, can be cleaved into short form under the digestion of the intestinal peptidase, OMA1, and the i-AAA proteolytic enzyme, YME1L, to induce mitochondrial fragmentation and fission in the membrane space (19).

### Mitochondrial Fission Proteins

Mitochondrial fission in mammalian cells is manipulated by DRP1, FIS1, mitochondrial fission factor (MFF), and mitochondrial dynamic proteins of 49 and 51 kDa (MiD49/51) as shown in Figure 2 (24). DRP1, a GTPase located in the cytoplasm, mediates mitochondrial fission at the outer membrane (28). DRP1 is encoded by the *DNM1L* gene and contains a GTPase region, an intermediate region, a polytropic region, and a GTPase effector region from the N-terminal to the C-terminal, which are involved in the physical constriction of the mitochondria (the early step of fission) (29). Notably, DRP1 needs to bind with other receptor proteins, such as FIS1, to embed on the outer membrane of the mitochondria because of its lack of lipid-interacting hydrophobic transmembrane domain (30). However, FIS1 depletion has minimal effect on the transfer of DRP1 to the mitochondria in mammalian cells (31). A multitude of receptors is involved in the recruitment of DRP1 to the mitochondria to trigger fission (32). Mid49/51 are involved in DRP1 translocation in this fission machinery (30). In addition, DRP1 activity is regulated by PTMs, such as acetylation and phosphorylation (32). DRP1 activity is modulated by two serine phosphorylation sites with opposing functions; that is, DRP1 activity can be activated by phosphorylation at serine 616 but inhibited by phosphorylation at serine 637 (33). Each serine phosphorylation is catalyzed by a different kinase and phosphatase; thus, mitochondrial fission is linked to key cellular processes (Table 1). For instance, the DRP1 phosphorylation at serine 616 mediated by the mitotic initiator, cyclin B1–cyclin-dependent kinase (CDK1), links mitochondrial fission to cell division (34). The phosphorylation mediated by calcium-calmodulin-dependent kinase (CamK) coordinates fission to intracellular calcium (35). The serine ratio between the 616 and 637 sites modulates DRP1 activity and reflects the polymerized influence of several kinases and phosphatases (36). DRP1 activity is also modulated by the ubiquitin ligases, membrane-associated RING-CH protein 5 and small ubiquitin-like modifier type 1 (37). DRP1 acetylation regulates the activity of itself and contributes to metabolic stress-associated cardiomyocyte death and dysfunction (38). Previous studies depicted that DRP1 forms a dimer or tetramer under basic conditions and further self-assembles into a larger polymer structure in the fission process.

Multiple PTMs of human mitochondrial proteins, such as phosphorylation (in MFN1, MFN2, and OPA1), acetylation (in MFN1, MFN2, and OPA1), methylation (in MFN1 and OPA1), and ubiquitination (in MFN1, MFN2, and OPA1) have been detected by mass spectrometry-based proteomics (39). However, the modulation of PTM in mitochondrial fusion proteins is largely uncharacterized compared with that in DRP1. For instance, the PTMs of MFN2 (phosphorylation and ubiquitination) are observed in hearts with cardiomyopathy. PINK1-dependent MFN2 phosphorylation induces Parkin translocation to the outer mitochondrial membrane upon membrane depolarization, which subsequently promotes Parkin-mediated MFN2 ubiquitination in adult cardiomyocytes (40). Also, Parkin-mediated MFN2 ubiquitination leads to MFN2 degradation, which results in the selective removal of damaged mitochondria by mitophagy in adult cardiomyocytes (41).

### Mitochondrial miRNAs

Apart from proteins, mitochondrial miRNAs (mitomiRs) modulate the translational activity of the mitochondrial genome and mitochondrial function (42). Mitochondrial fission/fusion can also be regulated by mitomiRs. Notably, miR-146a, miR-34a, and miR-181a may regulate mitochondrial dynamics by targeting Bcl-2 (42). Other mitomiRs can also directly target mitochondrial fusion/fission proteins. miR-484 suppresses FIS1-mediated fission and apoptosis in cardiomyocytes by decreasing FIS1 expression. Mitochondrial fission is also suppressed by the miR-30-mediated downregulation of DRP1 and p53 (43).

### Regulators of Mitochondrial Dynamics

Mitochondrial fusion and fission can also be mediated by peroxisome proliferator-activated receptor γ co-activator 1α (PGC-1α), which is a modulator of mitochondrial fusion by acting as a transcriptional coactivator of MFN2 (44, 45). The assembly of fission apparatus also needs the assistance of the ER directly in contact with the mitochondria to form a microdomain that facilitates the assembly of DRP1, MEF, and proapoptotic proteins (46). The lipids produced by mitochondrial phospholipase D, especially phosphatidic acid, guide mitochondrial dynamics (47).

The cooperation of mitochondrial fusion and fission maintains the fundamental integrity and normal functioning of the mitochondria, including energy metabolism, ROS generation, and apoptosis regulation (48). Fusion favors mitochondrial interconnection, mitochondrial DNA mixing, signal transduction, and metabolite exchange (49). Mitochondrial fission facilitates the elimination of damaged mitochondria by dividing the mitochondria into daughter mitochondria to maintain the normal function of the mitochondria (50). However, the perturbation of mitochondrial fusion and fission breaks their balance and consequently leads to the accumulation of damaged and non-functional mitochondria (48).

Mitochondrial dynamics play an important role in the morphology, function, and distribution of mitochondria. Fusion and fission regulate mitochondrial shape, length, and number. The balance between mitochondrial fusion and fission controls mitochondrial morphology. Mitochondrial shape affects the ability of cells to distribute their mitochondria to specific subcellular locations. Fusion and fission allow the mitochondrial exchange of lipid membranes and intramitochondrial content, which is crucial for maintaining the health of a mitochondrial population (51). For instance, MFN1 and MFN2 ablation in fibroblasts induce reduced respiratory capacity and great heterogeneity in mitochondrial shape and membrane potential (52).

## Endothelial Function and the Mitochondria

The proper function of the mitochondria in the arterial wall is critical in all atherogenesis-related key cell types, including ECs, VSMCs, and macrophages, which are responsible for massive lipid storage via phagocytosis, as well as pro-inflammatory status maintenance in a lesion (53). Normal endothelium is a dynamic organ that regulates vascular tone by balancing the production of vasodilators and vasoconstrictors in response to a variety of stimuli (54). The endothelial mitochondria act as critical signaling organelles that play a crucial role in endothelial function, including subcellular location, dynamics, biogenesis, mitophagy, autophagy, ROS; therefore, mitochondrial dysfunction facilitates atherosclerosis development (55, 56). Endothelial dysfunction is a pathological condition characterized by an imbalance between substances with vasodilating, antimitogenic, and antithrombogenic properties (endothelium-derived relaxing factors) and substances with vasoconstricting, prothrombotic, and proliferative characteristics (endothelium-derived contracting factors) (57). ECs play important roles in the maintenance of vascular homeostasis by modulating vasodilation, platelet activation, and leukocyte adhesion (58). Therefore, the dysfunction of EC leads to increased vascular tension and atherosclerosis, followed by systemic hypertension, and increased incidence of ischemia and stroke. Moreover, mitochondrial dysfunction is involved in the formation of oxidative stress conditions in atherosclerosis, which facilitate inflammatory response and lesion development (59).

In pulmonary ECs, DRP-1 activation, which induces mitochondrial fission, stimulates angiogenesis by promoting cell proliferation and migration and inhibiting apoptosis (60). Endothelial dysfunction contributes to the development of nearly all vascular diseases (10). Even though ECs have low mitochondrial content, mitochondrial dynamics act as a pivotal orchestrator of EC homeostasis under normal conditions; damage in mitochondrial dynamics participates in endothelial dysfunction and diverse vascular diseases. Endothelial dysfunction leads to altered mitochondrial morphology, reduced network extent, and increased FIS1 protein expression compared with ECs from healthy volunteers (61).

## Vascular Smooth Muscle Cell Function and the Mitochondria

VSMCs are the main constitutive stromal cells of the vascular wall that engage in a variety of different structural and physiological functions (62). VSMCs are crucial components of blood vessels and the major determinants of vasotone (62). This critical and tightly regulated function is granted by the contractile phenotype of VSMCs. VSMCs can switch to a synthetic dedifferentiated phenotype characterized by increased proliferative and migratory capabilities in response to certain cues. The VSMC phenotypic switch is implicated in the pathogenesis of vascular diseases (63). During the progression of atherosclerosis, VSMCs are subjected to a phenotype switch that can internalize atherogenic LDL particles, such as oxidized LDL or desialylated LDL, for lipid accumulation to migrate to lesion sites (64, 65). Cells with lipid particle accumulation are recognized as “foam cells” and manifest as atherosclerotic plaques (66). The association between VSMCs and mitochondrial dysfunction in atherosclerosis has been discussed before.

Mitochondrial dysfunction characterized by decreased oxidative phosphorylation is a striking phenotype of VSMCs isolated from atherosclerosis (13). In addition, a multitude amount of energy and oxygen-free radicals are required for the impairment of nuclear and mitochondrial DNAs in VSMCs, which further promotes DNA damage, genomic instability, and mitochondrial damage (67). Mitochondrial fission and fusion also affect VSMC function (68). Mitochondrial fission is an integral process in cell migration, and controlling mitochondrial fission can limit VSMC migration and pathological intimal hyperplasia by altering mitochondrial energetics and ROS levels (69). For instance, mitofusin (MFN) 2 is an important suppressor of VSMC proliferation (70). In addition, the link between mitochondrial dynamics and VSMC senescence can be mediated by Krüppel-like factor 5 (Klf5), an essential transcriptional factor of cardiovascular remodeling. Klf5 downregulation induces VSMC senescence through eIF5a depletion and mitochondrial fission (71).

## Macrophage and Monocyte Function and the Mitochondria

Macrophage mitochondrial fission is essential for the continued removal of apoptotic cells and plays a protective role in advanced atherosclerosis (72). In macrophage-enriched murine atherosclerosis lesion areas, the level of dynamin-related protein-1 (DRP1) is downregulated and MFN2 is upregulated as the lesion progresses. Inhibiting macrophage mitochondrial fission results in a dramatic increase in the necrotic core area and the accumulation of apoptotic cells in the advanced stage of atherosclerosis; thus, macrophage mitochondrial fusion/fission could be a potential therapeutic target to prevent lesion necrosis and stabilize advanced plaques (73). Human CD14+ monocytes exhibit reduced mitochondrial fission and increased mitochondrial fusion for metabolic adaptation upon lipopolysaccharide stimulation. Notably, mitochondrial dynamics affect the inflammatory responses of CD14+ monocytes.

## Mitochondrial Dynamics Imbalance

Many studies have pointed out the beneficial effects of mitochondrial fusion in oxidative phosphorylation. Mitochondrial fusion maintains normal mitochondrial function by protecting from mitochondrial DNA loss and maintaining the synthesis of mitochondrial proteins (74). In addition, mitochondrial fusion events can attenuate the damage of DNA and protein contents and restore damaged mitochondria by “functional complementation” (75). Mitochondrial fusion damage can lead to increased mitochondrial fission and fragmentation, which induce oxidative phosphorylation and cell apoptosis attenuated by mitochondrial division (76). For example, DRP1 gene mutation in mice can damage mitochondrial function and induce mitophagy, which contribute to heart enlargement and failure (77). Mitochondrial division inhibitor 1 (Mdivi-1) is a selective cell-permeable inhibitor of mitochondrial division DRP1 and mitochondrial division dynamin I. Mdivi-1 attenuates mitophagy and enhances apoptosis. Also, DRP1 inhibition with Mdivi-1 protects the injured heart and brain from ischemia (78, 79). Mitochondrial fission seems harmful in this perspective; however, the deletion of myocardial DRP1 gene can lead to division disorders, which result in dysfunctional mitochondria and ultimately lead to heart failure and death (80). Mitochondrial dynamics proteins have been genetically alerted in vascular cells. For example, in VSMCs, the overexpression of the phospho-deficient mutation, MFN2-S442A, increases the inhibitory effects of MFN2 on cell proliferation, as well as neointimal hyperplasia and restenosis, in rat carotid artery balloon injury model (70).

Excessive mitochondrial fragmentation often occurs in most vascular diseases and thus could be a promising therapeutic target for these diseases (81). The promotion of mitochondrial fusion and the inhibition of mitochondrial fission guide the different fates of the heart (82). MFN2 upregulation, besides DRP1 downregulation, maintains mitochondrial function through the elimination of excessive mitochondrial fragmentation (83). Mitochondrial fusion promoter, M1 (2 mg/kg), as an intervention in rat ischemia–reperfusion (I/R), reduces infarct size and exerts a beneficial effect toward ischemia (84). This result demonstrated that increased mitochondrial fusion brings about a beneficial impact on myocardial I/R injury.

However, excessive mitochondrial fusion causes serious diseases (85). Point mutation in mitochondrial carrier protein, SLC25A46, promotes the protein's rapid degradation and the stable recruitment of MFN2 and MFN1 complexes to the mitochondria and ultimately leads to over-fusion and the phenotype of cerebellopontine hypoplasia (86). Additionally, excessive mitochondrial fusion results in elevated oxidative stress and abnormal Ca^2+^ homeostasis, which eventually cause arrhythmia, particularly atrial fibrillation (87). Therefore, the balance between mitochondrial fusion and fission plays a vital role in the normal function of the vascular system.

## Pathogenesis of Vascular Diseases

Structurally, the normal artery is composed of three layers (88). The inner layer lined by a monolayer of ECs is closely contacted with blood; the middle layer composed of VSMCs is located at the complex extracellular matrix; and the outer layer of arteries is composed of mast cells, nerve endings, and microvessels (89). Direct contact with blood makes ECs especially vulnerable to damages caused by molecules (90). ECs act as ideal protection because they sense alterations in external stimuli and directly respond or transmit signals; EC dysfunction leads to the pathogenesis of almost all types of vascular diseases (91). Despite the low mitochondrial content of ECs, mitochondrial dynamics is a key endothelial homeostasis coordinator under normal conditions (10).

Atherosclerosis is the leading cause of vascular diseases and responsible for almost 50% of all cardiovascular deaths, and the mechanism of atherosclerosis has been well studied. Its pathogenesis comprises respective mechanisms during different disease stages (8). It is initiated through atherosclerotic lesion formation with a phenotype of endothelial dysfunction (92). The endothelium provides the functional link between blood circulation and the vessel wall. Local disturbance to the arterial endothelium leads to cell activation, which promotes the recruitment of circulating immune cells and increases permeability for circulating lipoprotein particles (93). Low-density lipoprotein (LDL), especially in its modified atherogenic form, is the main source of lipids that accumulate in the arterial wall (94). Several studies have confirmed the close association between the mitochondria and the different stages of atherosclerosis (95).

## Mitochondrial Dynamics and Vascular Diseases

Multiple factors are responsible for vascular diseases, including the infiltration, differentiation, and transformation of monocytes to active lipid foam cells, as well as VSMC migration to the intima (96). ROS production in the mitochondria is a key factor in vascular diseases (97).

**Figure 3:**
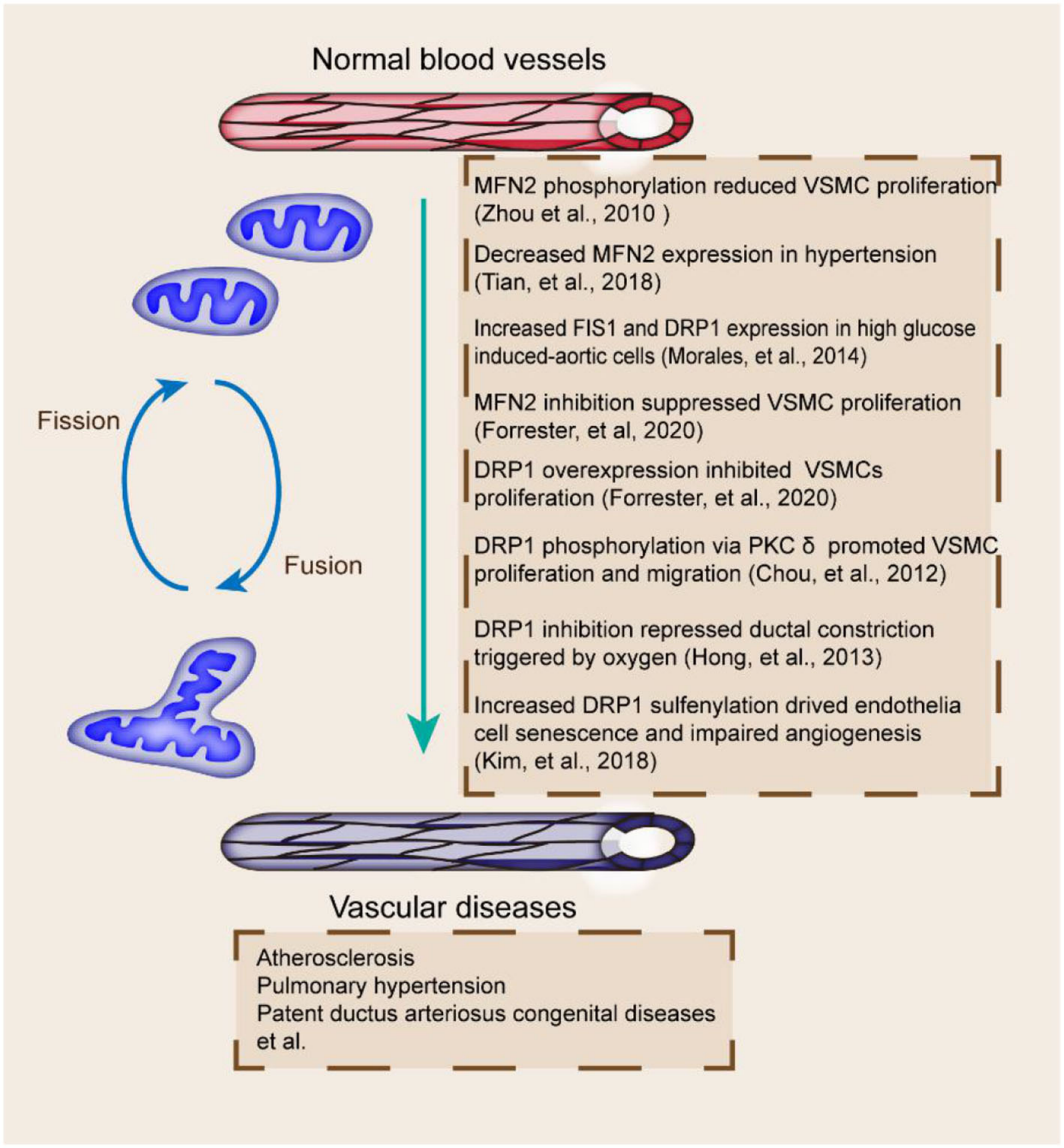
Mitochondrial fusion and fission proteins and vascular diseases. Fission and fusion imbalances are related to multiple vascular abnormalities, including MFN2 decrease and PTM, DRP1 inhibition and phosphorylation, and dysregulated FIS1 and DRP1 (70, 99, 104, 106, 114, 118, 122). The effects of proteins involved in the pathogenesis of vascular diseases are included.

Mitochondrial dynamics plays an important role in the progression of vascular diseases (Figure 3). Mitochondrial fragmentation and FIS1 expression are increased in patients with type 2 diabetes (98). DRP1 and FIS1 accumulate in human aortic ECs after high glucose treatment (99). Alterations in mitochondrial dynamics are correlated with the production of mitochondrial ROS, which affects the pathogenesis of vascular diseases (100). FIS1 and DRP1 inhibition can block the production of mitochondrial ROS and mitochondrial network; hence, mitochondrial fission has a vital role in vascular diseases (37).

The coordination of mitochondrial fusion and fission is essential for the maintenance of mitochondrial quantity and quality. Mitochondrial fragmentation occurs in vascular diseases (Table 2). Functional ductus arteriosus closure, initially induced by oxygen-dependent vasoconstriction shortly after birth, is dependent on mitochondrial fission (97). DRP1 perturbation is associated with endothelial dysfunction (101). DRP1-mediated mitochondrial fission exerts a critical function in the acute constriction of the ductus arteriosus to O^2^ and participates in the subsequent anatomic closure of the ductus arteriosus (102). Mitochondrial fission also seems indispensable for angiogenesis in ECs (103). The loss of protein disulfide isomerase active 1 in ECs induces mitochondrial fragmentation and mitochondrial ROS elevation by increasing Cys644 sulfenylation and DRP1 activity, which impair endothelium-dependent vasorelaxation and angiogenesis (104). DRP1 depletion in mice also leads to defective efferocytosis and has pathologic consequences in the thymus after dexamethasone treatment and in the advanced atherosclerotic lesions of fat-fed LDLR^−/−^ mice (105). DRP1 overexpression or MFN2 inhibition also leads to endothelial dysfunction and the inhibition of VSMC proliferation (106). The decreased expression of MFN1 and MFN2 promotes atherosclerosis in animal models (107).

**Table 2:** Role of mitochondrial dynamics protein in vascular diseases.

**Mediator**	**Cellular phenotype**	**Vascular disease involving abnormalities of mitochondrial dynamics**
**Fusion mediators**		
Mitofusin-1	VSMC proliferation and migration	Atherosclerosis
Mitofusin-2	VSMC proliferation and migration, proliferation of pulmonary artery smooth muscle cells,	Pulmonary arterial hypertension, arterial restenosis, Atherosclerosis, arterial restenosis
Optic atrophy 1		Hypertension
**Fission mediator**		
DRP1	VSMC proliferation, phenotypic alterations of VSMCs, apoptosis	Patent ductus arteriosus, pulmonary arterial hypertension, Atherosclerosis,
FIS1	Increased FIS1 in Endothelial dysfunction,	

The functions of MFN1 and MFN2 in ECs have also been addressed (108). Interestingly, the expression of MFNs could be stimulated in ECs when exposed to the angiogenic mitogen, vascular endothelial growth factor (VEGF) (109). The knockdown of MFN1 and MFN2 prevents the endothelial migration and differentiation induced by VEGF (110). Additionally, the diverse roles of MFNs in ECs were measured (111). MFN2 inhibition exclusively attenuates the production of basal and stress-induced ROS (96). MFN1 ablation particularly blocks VEGF signal transduction and suppresses NO production (23). Interestingly, the role of MFNs in vascular pathology is tightly related to metabolic stress (112).

Mitochondrial fission is indispensable for VSMC proliferation and migration, as well as pathophysiological processes, such as the premature closure of open arterial ducts and pulmonary hypertension (113). On the occasion of oxidative stress and angiotensin II stimulation, activated protein kinase C δ phosphorylates DRP1, which leads to mitochondrial fission and ROS-stimulated VSMC proliferation and migration (114). Therefore, the pharmacological inhibition of DRP1 could be used as a therapeutic target.

Emerging evidence implied that alteration in mitochondrial dynamics is accompanied by acute I/R. Several researches have observed that reduced OPA1 and MFN2 and increased DRP1 in cardiomyocytes simulate I/R. I/R stimulation in HL-1 cells induces mitochondrial fission through DRP1; the transfection of the fusion protein or DRP1 dominate-negative mutant protects from I/R injury (115). Moreover, OPA1 mild overexpression transgenic mice are resistant to muscular atrophy and I/R damage in the heart and brain. In spite of the well-known impact of mitochondrial fission and fusion balance on cardiac I/R injury, no study has shown a direct indication of their potential role in ischemic myopathy in peripheral artery disease.

Mitochondrial fusion and fission are also implicated in the abdominal aortic aneurysm (AAA). Angiotensin II stimulation (one of the main methods to induce AAA) in cultured rat aortic VSMCs induces mitochondrial fission. DRP1 expression was enhanced in human AAA samples compared with age-matched healthy controls (116). Furthermore, DRP1 inhibition protects from AAA development, as assessed by the diameters of the abdominal aorta as well as histological observation. Protection against AAA by DRP1 inhibition is accompanied by reduced stress response and senescence. Therefore, DRP1-mediated mitochondrial fission potentially stimulates the proinflammatory phenotypic alterations of VSMCs and contributes to the pathogenesis of AAA development.

As an impeditive vascular disease, pulmonary arterial hypertension is induced by several factors, including disordered oxygen sensing and dysregulated mitochondrial dynamics in pulmonary artery smooth muscle cells (117). Pulmonary arterial hypertension is believed to be contributed by excessive cell proliferation and impaired apoptosis accompanied by vasoconstriction, inflammation, and thrombosis (92). Pulmonary arterial hypertension is accompanied by reduced MFN2 and excessive DRP1 caused by increased hypoxia inducible factor 1 alpha (HIF-1α) activation and decreased PGC-1α activity (118). MFN2 mediates the proliferation of pulmonary artery smooth muscle cells in hypoxic pulmonary hypertension via the PI3K/Akt pathway (119). HIF-1α activation induces DRP1-dependent mitochondrial fission and an imbalance in fusion and fission in normal pulmonary artery smooth muscle cells (120). The decrease in MFN2 in pulmonary arterial hypertension leads to mitochondrial fragmentation and proliferation (121).

DRP1 inhibition represses the ductal constriction triggered by oxygen (122). Oxygen induces the PTM of DRP1 mediated by cyclin B1-CDK1 and CamK to trigger mitochondrial fission (123). Although the continuous inhibition of DRP1 impedes structural closure in an *in vitro* model of human open ductus arteriosus, the previous study still has not elucidated whether damaged mitochondria result in spontaneous patent ductus arteriosus (122).

MiRNA expression alteration contributes to ischemic heart disease by regulating the expression of various key mitochondrial elements involved in cell survival and death. MiR-762 and miR-210 are elevated whereas miR-1 is downregulated in myocardial infarction. miR-762 knockdown alleviates myocardial I/R injury in mice. The upregulation of miR-15/16 and miR-195 modulate cardiomyocyte survival and myocardial infarction by inhibiting ATP levels and inducing mitochondrial fusion. In addition, miR-15 inhibition protects against I/R injury *in vivo* by targeting pyruvate dehydrogenase kinase 4 and serum/glucocorticoid-regulated kinase 1, which are responsible for mitochondrial function and apoptosis, respectively (124). miRNAs also regulate foam cell formation and subsequent plaque formation. miR-302a suppresses foam cell formation, which would aggravate atheromatic plaque by increasing the activity of ABCA1, which induces the efflux of cholesterol out of macrophages.

## Mitochondrial Dynamic Regulatory Proteins as Therapeutic Targets

**Figure 4:**
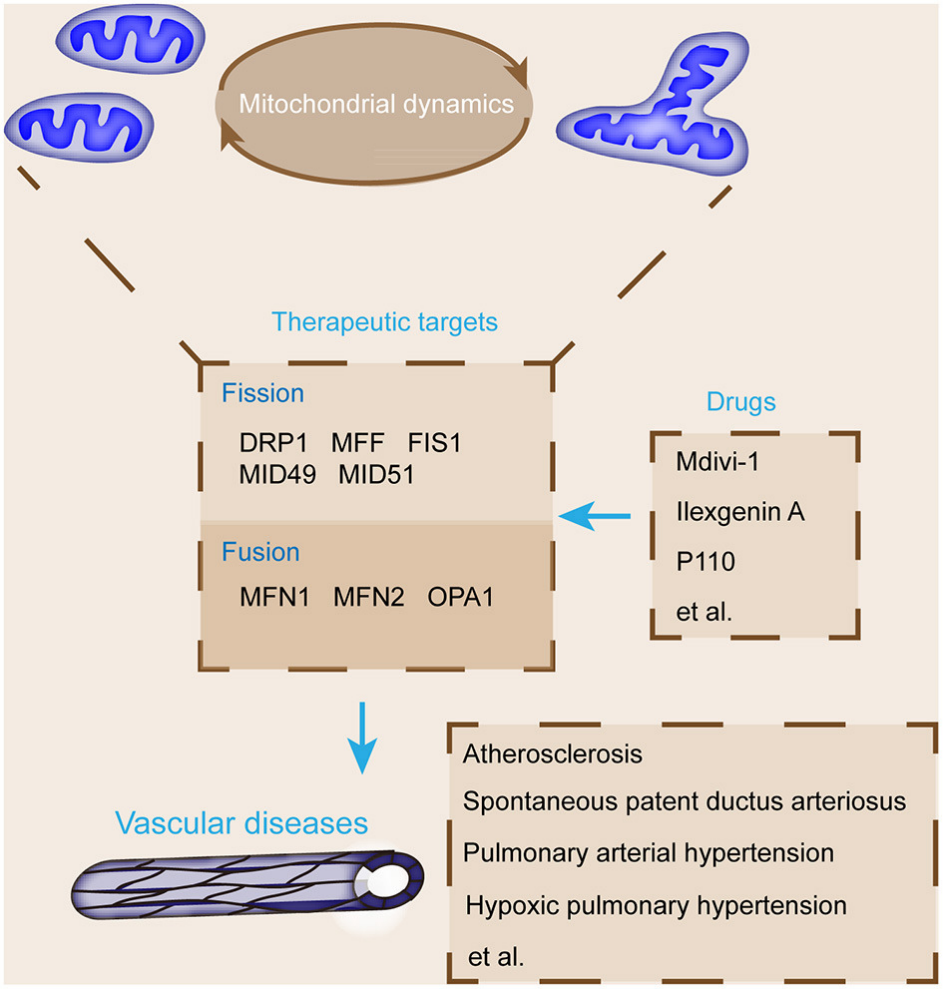
Summary of key mitochondrial dynamic regulatory proteins and major pharmacological agents that target these proteins for the treatment of vascular diseases. The targeting agents of mitochondrial fusion and fission proteins, such as DRP1 and MFN1, protect from vascular diseases, including pulmonary arterial hypertension, atherosclerosis, and ductus arteriosus closure.

Supporting materials relate mitochondrial fusion and fission to vascular diseases; emerging studies elucidated the protective effects of mitochondrial fusion and fission modulators on vascular diseases (Figure 3) (125). The pharmacological inhibition of DRP1 relieves plaque formation and lessens the accumulation of macrophages in the plaques of the ApoE^−/−^ mouse model of carotid artery injury induced with wire (126). DRP1 seems to be a promising novel therapeutic target for atherosclerosis (106). As a selective cell-permeable inhibitor of mitochondrial division, Mdivi-1 treatment can dramatically reduce atherosclerotic lesion formation in streptozotocin-induced diabetic ApoE^−/−^ mice (127). Mdivi-1 inhibits VSMC proliferation and migration through the attenuation of ROS production and DRP1 phosphorylation (128). Moreover, the anti-proliferation effect of Mdivi-1 is dependent on G2/M cell cycle arrest and independent on cyclin B1/CDK1-mediated DRP1 phosphorylation in arterial smooth muscle cells (118). Another example is ilexgenin A, a novel pentacyclic triterpenoid that exerts anti-atherosclerotic activity to reduce atherosclerosis in apolipoprotein E-deficient mice. Ilexgenin A hinders mitochondrial fission and induces DRP1 degradation dependent on Nrf2-induced proteasome subunit beta 5 in ECs, which contribute to the restraint of mitochondrial fission and thus relieve endothelial dysfunction (129). These findings provide the theoretical basis for the future development of ilexgenin A as a potential agent for atherosclerosis treatment. Mdivi-1 or congeners could also be used to maintain ductus arteriosus patency in infants awaiting congenital heart surgery (120). In addition, Mdivi-1 administration facilitates premature senescence and destroys the angiogenic function of human umbilical cord vein ECs by promoting the production of mitochondrial ROS and reducing autophagy flux (78). Therefore, DRP1 may be a promising therapeutic target for vascular repair (Figure 4).

## Concluding Remarks

Mitochondrial dynamics is associated with the pathogenesis of various vascular diseases and provides potential therapeutic targets (12, 56, 97, 98). Further identification prior to trial on potential therapeutic agents related to mitochondrial dynamics is indispensable to determine proper molecular targets and definitions and confirm the optimal and effective doses for mitochondrial fusion and fission modulators (130, 131). Extra modulators for mitochondrial fusion and fission are required (121, 132). For example, a recently designed inhibitor, P110, can inhibit DRP1 activation and fission by blocking the interaction between DRP1 and FIS1 (133).

In spite of the development of pharmacological agents that target fusion and fission for the prevention and treatment of vascular diseases, several obstacles remain to be solved to achieve this goal (87, 103, 104). First, the therapeutic agent needs to have specificity to target the organ and ascertain the duration time (134). Second, mitochondrial fusion and fission are vital for the proper functioning of the mitochondria and normal cells; hence, the manipulation of fusion and fission might have detrimental effects on normal cells. Besides, the application of such therapeutic agents is limited to temporary acute conditions rather than chronic conditions. Moreover, off-target effects should also be minimized. The off-target effects of these pharmacological agents are often caused by the recognition of the binding sites of the drug by other biomacromolecules, including receptors, enzymes, ion channels, transporters, and genes. This occurrence is still an important issue in the study of vascular related inhibitors.

In addition to energy metabolism, the mitochondria have multidimensional influence on cells and the vascular system. For example, the regulation of mitochondrial calcium homeostasis and mitophagy can affect vascular development and functional maintenance, but related molecular mechanisms still need further theoretical support (71, 105, 106). Whether mitochondrial homeostasis can cooperate with other mitochondrial functions to jointly affect vascular development and disease, as well as how key proteins play roles in this dynamic interaction process, needs further study. Mitochondrial dynamic regulatory proteins, such as FIS1, have a variety of functions. Their effects on vascular function still need to be studied in depth if these proteins will be used as therapeutic targets for vascular diseases.

## Author Contributions

YY and YL conceptualized, wrote the manuscript, and created Figures. YL, XC, and K-DR contributed to the writing of the manuscript. YY, XC, and YL reviewed and modified the manuscript. All authors approved the final version of the manuscript.

## Funding

This work was supported by the National Natural Science Foundation of China (No. 31900502), the Henan Medical Science and Technology Joint Building Program (No. LHGJ20190236), and Key scientific Research project of Henan Universities (No. 21A310027).

## Conflict of Interest

The authors declare that the research was conducted in the absence of any commercial or financial relationships that could be construed as a potential conflict of interest.

## Publisher's Note

All claims expressed in this article are solely those of the authors and do not necessarily represent those of their affiliated organizations, or those of the publisher, the editors and the reviewers. Any product that may be evaluated in this article, or claim that may be made by its manufacturer, is not guaranteed or endorsed by the publisher.
